# 

**Published:** 1917-04

**Authors:** 


### THE BRISTOL
### Medico-Chirurgical Journal.

A JOURNAL OF THE MEDICAL SCIENCES FOR THE
WEST OF ENGLAND AND SOUTH WALES.

*PUBLISHED UNDER THE AUSPICES OF*

*THE BRISTOL MEDICO-CHIRURGICAL SOCIETY.*

EDITOR:
P. WATSON-WILLIAMS, M.D.

WITH WHOM ARE ASSOCIATED
J. MICHELL CLARKE, M.A., M.D., LL.D., J. LACY FIRTH, M.S., M.D.,
AND JAMES SWAIN, C.B., M.S., M.D.

ASSISTANT-EDITOR:
J. A. NIXON, B.A., M.D.

ACTING EDITORIAL SECRETARY:
W. A. SMITH, M.A., M.B.

---

"Scire est nescire, nisi id me

Scire alius sciret."

---

528955

*VOL. XXXV.*

BRISTOL: J. W. ARROWSMITH LTD.
LONDON: SIMPKIN, MARSHALL, HAMILTON, KENT & Co. LIMITED.
1917.

J.W. Arrowsmith Ltd

Publishers Bristol

### CONTENTS OF VOLUME XXXV.

*Page*

Introduction to a Discussion on the Diagnosis of Tuberculosis. By LATIMER JAMES SHORT, M.D., B.S. Lond., M.D. Brist., D.P.H. Cantab. .. .. .. .. .. .. .. .. 1

Notes on Plague in Bristol in 1916. By LIEUT.-COLONEL D. S. DAVIES, R.A.M.C. .. .. .. .. .. .. .. 16

Chronic Appendicitis in its Relation to Dyspepsia. By H. GOODWYN, F.R.C.S. Ed. .. .. .. .. .. .. .. .. 18

Fatigue Induced by Labour. By A. F. STANLEY KENT, M.A., D.Sc. Oxon. .. .. .. .. .. .. .. .. 47

Gunshot Wounds of Peripheral Nerves. By LIEUT.-COLONEL J. MICHELL CLARKE, R.A.M.C. (T:), M.D., F.R.C.P. .. .. 57

The Prevention of Sepsis in War Wounds, with special reference to the Carrel-Dakin Method. By LIEUT.-COLONEL JAMES SWAIN, R.A.M.C., C.B., M.S., M.D. Lond., F.R.C.S. .. .. .. 68

Acute Pancreatitis, with special reference to its treatment, and a record of three cases presenting unusual features. By MAJOR CHARLES A. MORTON, F.R.C.S· .. .. .. .. .. 80

Rectal Diverticula as a Causative Factor in Pelvic Inflammation in Women. By MAJOR WALTER C. SWAYNE, M.D. Lond. and Brist. 91

War Surgery in an Indian General Hospital in Mesopotamia. By PERCIVAL S. CONNELLAN, I.M.S. .. .. .. .. .. 113

REVIEWS OF BOOKS .. .. .. .. .. 24, 96, 129

EDITORIAL NOTES .. .. .. .. .. .. .. 22, 107

NEW DRUGS AND PREPARATIONS FOR THE SICK .. .. .. 32

OBITUARY NOTICES .. .. .. .. .. 35, 110, 136

### Bristol Medico-Chirurgical Society.

### President.

F. H. EDGEWORTH, M.A., M.D. Cantab., D.Sc. Lond.

### President-Elect.

FREDERICK LACE, F.R.C.S. Eng., L.R.C.P. Lond.

### Committee.

*Ex-Officio.*
- GEORGE PARKER, M.A., M.D. Cantab. (Retiring President).
- P. WATSON-WILLIAMS, M.D. Lond. (Editor of *Journal*).
- C. K. RUDGE, M.R.C.S. (Hon. Librarian).
- W. ALEXANDER SMITH, M.A., M.B. Oxon. (Acting Editorial Secretary).

*Elected.*

R. SHINGLETON SMITH, M.D., B.Sc. Lond., Ex-President.
I. WALKER HALL, M.D. Vict.
J. LACY FIRTH, M.D., M.S. Lond.
J. O. SYMES, M.D. Lond.
L. E. V. EVERY-CLAYTON, M.D., B.S. Lond.
R. G. P. LANSDOWN, M.B., B.S. Durh.
F. H. EDGEWORTH, M.A., M.D. Cantab., D.Sc. Lond.

### Acting Honorary Secretary.

A. L. FLEMMING, M.B., Ch.B. Bristol.

Medical Practitioners wishing to join the Society are requested to communicate with the Acting Hon. Secretary, Dr. A. L. FLEMMING, 48 Pembroke Road, Clifton. The Annual Subscription is 21/-.

---

*Extracts from Journal By-laws.*

4.—The *Journal* shall be delivered free to Subscribers and also to Members of the Society whose subscriptions are not in arrear.

6.—The Annual Subscription to the *Journal* for those who pay before the 1st of July shall be 5/-, post free, or 6/- if paid after that date.

---

Communications intended for insertion in the *Journal* should be sent to the Editor, Dr. WATSON-WILLIAMS, 2 Rodney Place, Clifton, Bristol.

Books for Review and Exchange Journals should be sent to the Assistant-Editor, *Bristol Medico-Chirurgical Journal*, Medical Library, The University, Bristol.

Communications referring to the delivery of the *Journal* should be sent to the Acting Editorial Secretary, Dr. W. A. SMITH, 70 Pembroke Road, Clifton, Bristol, to whom Subscriptions are to be paid *in advance*.

Cases for Binding Vols. I—XXXIV are now ready, and may be obtained, price 10*d*. each (postage 2*d*. extra), upon application to J. W. ARROWSMITH LTD., Quay Street, Bristol; or the *Journal* will be bound, including Case, for 1/9 per volume.

Owing to the war, no March or September issue of the *Journal* was published, so that Vol. XXXIV consisted of the two special parts numbered 130, 131.

### Bristol Medico-Chirurgical Society.

### PAST PRESIDENTS.

1874—75. FREDERICK BRITTAN, M.D., B.A. Dub.
1875—76. ROBERT WILLIAM COE.
1876—77. SAMUEL MARTYN, M.D. Ed.
1877—78. AUGUSTIN PRICHARD, M.D. Berlin.
1878—79. HENRY EDWARD FRIPP, M.D. Würzburg.
1879—80. WILLIAM MICHELL CLARKE.
1880—81. JOSEPH GRIFFITHS SWAYNE, M.D. Lond.
1881—82. EDWARD LONG FOX, M.D. Oxon.
1882—83. JAMES GEORGE DAVEY, M.D. St. And.
1883—84. WILLIAM JOHNSTONE FYFFE, M.D., B.A. Dub.
1884—85. GEORGE FORSTER BURDER, M.D. Aberd.
1885—86. WILLIAM HENRY SPENCER, M.D., M.A. Cantab.
1886—87. FRANCIS POOLE LANSDOWN.
1887—88. LEMUEL MATTHEWS GRIFFITHS.
1888—89. ROBERT SHINGLETON SMITH, M.D., B.Sc. Lond.
1889—90. NELSON CONGREVE DOBSON, Ch.M. Bristol.
1890—91. SAMUEL HENRY SWAYNE.
1891—92. FRANCIS RICHARDSON CROSS, M.B. Lond., LL.D. Bristol.
1892—93. EDWARD MARKHAM SKERRITT, M.D., B.S., B.A. Lond.
1893—94. JAMES GREIG SMITH, M.B., C.M., M.A. Aberd., F.R.S.E.
1894—95. ALFRED JAMES HARRISON, M.B. Lond.
1895—96. ARTHUR WILLIAM PRICHARD.
1896—97. ALFRED EDWARD AUST LAWRENCE, M.D., C.M. Aberd.
1897—98. JOHN EDWARD SHAW, M.B., C.M. Ed.
1898—99. ROBERT ROXBURGH, M.B. Ed.
1899—00. WILLIAM HENRY HARSANT.
1900—01. DAVID SAMUEL DAVIES, M.D. Lond.
1901—02. BARCLAY JOSIAH BARON, M.B., C.M. Ed.
1902—03. GEORGE MUNRO SMITH, M.D. Bristol.
1903—04. JAMES PAUL BUSH, C.M.G., Ch.M. Bristol.
1904—05. REGINALD EAGER, M.D. Lond.
1905—06. JOHN DACRE.
1906—07. JAMES TAYLOR.
1907—08. HENRY WALDO, M.D., C.M. Aberd.
1908—09. JOHN MICHELL CLARKE, M.D., M.A. Cantab.
1909—10. JAMES SWAIN, M.D., M.S. Lond.
1910—11. BERTRAM MITFORD HERON ROGERS, M.D., B.Ch., B.A. Oxon.
1911—12. CHARLES ALEXANDER MORTON.
1912—13. WALTER CARLESS SWAYNE, M.D., B.S. Lond. and Bristol.
1913—14. PATRICK WATSON-WILLIAMS, M.D. Lond.
1914—15. WILLIAM HARRY CHRISTOPHER NEWNHAM, M.A., M B. Cantab.
1915—16. GEORGE PARKER, M.A., M.D. Cantab.

1917.

---

### LIST OF SUBSCRIBERS

TO THE

### Bristol Medico-Chirurgical Journal.

---

### HONORARY MEMBERS OF THE SOCIETY.

| | |
|---|---|
| OWEN, SIR ISAMBARD, D.C.L., M.D. | Hereford House, Clifton. |
| DOBSON, N. C., Ch.M., F.R.C.S. ... | 16 College Road, Clifton. |
| GRIFFITHS, L. M., M.R.C.S. ... ... | 11 Pembroke Road, Clifton. |
| SMITH, R. SHINGLETON, M.D., B.Sc., F.R.C.P. ... ... ... ... ... ... | Deepholm, Clifton Park, Clifton. |

---

### SUBSCRIBERS.

*Those marked * are Members of the Society.*

| | |
|---|---|
| ACKLAND, J. McKNO ... ... ... ... ... ... | 1 Barnfield Crescent, Exeter. |
| *ACKLAND, W. R., M.D.S. Bristol ... ... ... | 5 Rodney Place, Clifton. |
| ADYE, W. J. A. ... ... ... .. ... ... ... | St. Margarets, Bradford-on-Avon, Wilts. |
| ALDOUS, GEORGE F. ... ... ... ... ... ... | Charlton House, Mannamead, Plymouth. |
| *ALEXANDER, D.A., M.B., Ch.B. Vict.... ... ... | 30 Berkeley Square, Clifton. |
| ALFORD, H. J., M.D. Lond. ... ... ... ... ... | 19 Park Street, Taunton. |
| *ALMOND, G. H. H., M.A., M.B., B.Ch. Oxon. | 6 Brock St., Bath. |
| *ASKINS, R.A., M.D., B.Ch., B.A.O. Dubl. ... | Guildhall, Bristol. |
| *AUBREY, T., M.B. Lond. ... ... ... ... ... | Bitton, near Bristol. |
| AVELINE, H. T. S., M.D. Durh. ... ... ... ... | Somerset Asylum, Taunton. |

| | |
|---|---|
| *BAKER, LILY A., M.D., B.Ch., B.A.O., R.U.I. ... | 8 Richmond Hill, Clifton. |
| *BALLANCE, H. STANLEY M.D. Lond. ... ... | 1 Royal Terrace, Weston-super-Mare. |
| BARBER, M. C., M.B., Ch.B. Bristol ... ... | Ingledene, High Street, Staple Hill, Bristol. |
| *BARKER, G. H., M.D., B.Sc. Lond. ... ... ... | 124 Redland Road, Bristol |
| *BARON, BARCLAY J., M.B., C.M Ed.... ... ... | 16 Whiteladies Road, Clifton. |
| BASSET, WALTER ... ... ... ... ... ... ... | 60 Stow Hill, Newport, Mon. |

### LIST OF SUBSCRIBERS.

| | |
|---|---|
| *Benson, J. R. ... ... ... ... ... ... ... ... | Fiddington House, Market Lavington, Wilts. |
| *Bergin, F. G. ... ... ... ... ... ... ... ... | 31 Oakfield Road, Clifton. |
| Bernard, C. ... ... ... ... ... ... ... ... | 704 Fishponds Road, Fishponds, Bristol. |
| Berry, F. C., M.D., B.Ch., B.A. Dub. ... ... | Locarno, Burnham, Somerset. |
| Berry, James, M.B., B.S. Lond. ... ... ... | 21 Wimpole Street, London, W., 1. |
| Biamonti, Signor Sebastiano ... ... ... ... | 27 Via Balbi, Genoa, Italy. |
| *Blacker, A. E.... ... ... ... ... ... ... ... | 20 Victoria Square, Clifton. |
| Blackett, J. F., M.D., B.S. Lond. ... ... ... | 10 South Stoke Road, Combe Down, Bath |
| *Blagg, Arthur F., M.D. Durh.... ... ... ... | 28 Caledonia Place, Clifton. |
| Bodman, J. Hervey, M.D. Lond. ... ... ... | 9 Whiteladies Road, Clifton. |
| *Bowker, G. E., M.D. Edin.... ... ... ... ... | 11 North Parade, Bath. |
| *Brasher, C. W. J., M.B., Ch.B. Bristol ... ... | 17 Oakfield Road, Clifton. |
| *Brew, R. H. ... ... ... ... ... ... ... ... | Chew Magna. |
| *Bristowe, H. C., M.D. Lond. ... ... ... ... | Wrington, Somerset. |
| *Brown, William, M.D., C.M. Glasg. ... ... | Park View, Fishponds, Bristol. |
| *Burdon-Cooper, J., M.D., B.Sc. Durh. ... ... | 12 The Circus, Bath. |
| Burroughs, E. F. H. ... ... ... ... ... ... | 27 Burlington Road, Redland, Bristol. |

| | |
|---|---|
| *Cadman, E. S. R., M.D., C.M. Edin. ... ... | Gordon House, Westham, Pevensey, Sussex. |
| *Cairns, F. J. ... ... ... ... ... ... ... ... | Devon House, Whitehall Road, Bristol |
| *Carling, A., M.A., M.B., B.C. Cantab. ... ... | 12 Great George Street, Bristol. |
| *Carter, T. M., M.D. Durh. ... ... ... ... | Burnley, Westbury-on-Trym. |
| *Carwardine, Thomas, M.B., M.S. Lond. ... | 16 Victoria Square, Clifton. |
| *Cave, Edward J., M.D. Lond. ... ... ... ... | 20 Circus, Bath. |
| *Charles, J. R., M.A., M.D., B.C. Cantab. ... | 12 Victoria Square, Clifton. |
| *Chitty, H., M.S. Lond. ... ... ... ... ... | 46 Pembroke Road, Clifton. |
| *Christofferson, P. E., M.B., Ch.B. Bristol .. | 8 Lansdown Place, Clifton. |
| Chute, Henry M. ... ... ... ... ... ... ... | King William's Town, S. Africa. |
| *Clarke, C., M.B., B.S. Lond. ... ... ... ... | Brompton Hospital, London, S.W. |
| *Clarke, John Michell, M.D., M.A. Cantab. | 28 Pembroke Road, Clifton. |
| *Clarke, R. C., M.B., Ch.B. Bristol ... ... ... | Amherst House, Clifton Park, Clifton. |
| Clowes, E. F. ... ... ... ... ... ... ... ... | Grantley, Wotton-under-Edge, Glos. |
| Connellan, Lieut. P. S., I.M.S. ... ... ... | c/o Grindlay & Co., 54 Parliament Street, London, S.W. |
| *Cooke, H. W. ... ... ... ... ... ... ... ... | Lindthorpe, Southville, Bristol. |
| *Coombs, Carey, M.D. Lond. ... ... ... ... | 3 Pembroke Road, Clifton. |
| *Cooper, William Russell ... ... ... ... | 13 Westfield Park, Redland, Bristol. |
| *Corfield, C. .. ... ... ... ... ... ... ... | 12 Pembroke Road, Clifton. |
| Cossham, W. R., M.D., C.M. Aberd.... ... ... | Wellesley House, Cirencester. |
| Cotton, William, M.D., C.M., M.A. Ed. ... | 231 Gloucester Road, Bristol. |
| Coulter, R. J., M.B. Dub. ... ... ... ... ... | Ferncliffe, Stow Park Crescent, Newport, Mon. |
| Cridland, A. B. ... ... ... ... ... ... ... | Salisbury House, Chapel Ash, Wolverhampton. |
| *Cross, F. R., M.B. Lond., LL.D. Bristol ... | Worcester House, Clifton. |
| *Crossman, F. W., M.B. ... ... ... ... ... ... | White's Hill, Hambrook, near Bristol. |
| *Crouch, C. Percival, M.B. Lond. ... ... ... | Villa Rosa, Weston-super-Mare. |

### LIST OF SUBSCRIBERS.

| | |
|---|---|
| *DACRE, JOHN ... ... ... ... ... ... ... ... ... | 14 Eaton Crescent, Clifton. |
| *DAVIES, D. S., M.D. Lond., D.P.H. Cantab. ... | 6 Lansdown Place, Clifton. |
| DAVIES, NAUNTON W., F.R.C.S. Ed. ... ... | Llandrindod Wells. |
| DENING, EDWIN ... ... ... ... ... ... ... ... | Manor House, Stow-on-the-Wold, Glos. |
| DEVINE, H., M.D., Lond. ... ... ... ... ... | Corporation Mental Hospital, Portsmouth. |
| *DIXON, T. B. ... ... ... ... ... ... ... ... ... | 134 Coronation Road, Bristol. |
| *DOWLING, E. A. G. ... ... ... ... ... ... ... | 5 Rodney Place, Clifton. |
| *DRAPES, T. L., M.B., B.C., B.A. Cantab. ... | St. Maur, Beaufort Square, Chepstow. |
| *DUCKETT, A. H., M.B. ... ... ... ... ... ... ... | Hapsford House, South Stoke Road, Combe Down, Bath. |
| DUNBAR, ELIZA L. WALKER, M.D. Zürich ... | 9 Oakfield Road, Clifton. |
| DYMOKE, F. ... ... ... ... ... ... ... ... ... | 21 Cotham Road, Bristol. |
| | |
| *EDGEWORTH, F. H., M.D., B.C., M.A. Cantab., D.Sc. Lond. ... ... ... ... ... ... ... ... ... | 4 Richmond Park Road, Clifton. |
| EDWARDS, C. D., B.A., M.D. Cantab. ... ... | The Corderies, Chalford, Glos. |
| ELDER, WILLIAM ... ... ... ... ... ... ... ... | 4 Trelawney Road, Cotham, Bristol. |
| ELLIOT BROS. LTD. ... ... ... ... ... ... ... | *Australian Pharmaceutical Notes & News*, O'Connell Street, Sydney, N.S.W. |
| *ELLIOTT, CHRISTOPHER, M.D., B.A. Dub. ... | 102 Pembroke Road, Clifton. |
| ELLIOTT, F. PERCY, M.B., C.M. Aberd. ... ... | 113 Grove Rd., Walthamstow, London, E., 17. |
| *ESKELL, B.C., M.B., Ch.B. Bristol ... ... ... | 26 Zetland Road, Bristol. |
| *EVERY-CLAYTON, L. E. V., M.D. Lond ... ... | Emsworth House, Clevedon, Somerset. |
| | |
| *FAILL, C. J. C. ... ... ... ... ... ... ... ... ... | 40 Princes Street, Bristol. |
| FARNFIELD, W. W. ... ... ... ... ... ... ... ... | Clive Vale, Gillingham, Dorset. |
| *FAWN, G. F., B.D.S. Bristol ... ... ... ... | 25a Whiteladies Road, Clifton. |
| *FELLS, A., M.B., C.M. Edin. ... ... ... ... | 10 Elton Road, Tyndall's Park, Bristol. |
| *FIRTH, J. L., M.D., M.S. Lond. ... ... ... ... | 8 Victoria Square, Clifton. |
| *FLEMMING, A. L., M.B., Ch.B. Bristol ... ... | 48 Pembroke Road, Clifton. |
| *FLEMMING C. E. S. ... ... ... ... ... ... ... | Manvers House, Bradford-on-Avon. |
| *FORTESCUE-BRICKDALE, J. M., M.A., M.D., B.Ch. Oxon. ... ... ... ... ... ... ... ... | 52 Pembroke Road, Clifton. |
| FOSS, E. V., M.D., Ch.B. Bristol ... ... ... | Clouds Hill House, St. George, Bristol. |
| *FRASER, FORBES ... ... ... ... ... ... ... ... | 5 The Circus, Bath. |
| FREEMAN, J., M.D. Brux. ... ... ... ... ... | Waterloo Villa, 105 Queen's Road, Clifton. |
| | |
| *GALPIN, P. A., M.D., B.S. Durh. ... ... ... | 170 Browning Road, Manor Park, London, E., 12. |
| GARLAND, E. B., M.B., C.M. Edin. ... ... ... | 114*a* Hampton Road, Redland, Bristol. |
| *GERRISH, D. S., M.R.C.S. Lond.... ... ... ... | 328 & 330 Church Road, St. George, Bristol. |
| *GIBBS, A. N. GODBY ... ... ... ... ... ... ... | 52 Whiteladies Road, Clifton. |
| *GRATTE, C. BROOKE ... ... ... ... ... ... ... | 18 Gold Tops, Newport, Mon. |
| *GREEN, T. A., M.D., C.M. Edin. ... ... ... | 33*a* Whiteladies Road, Clifton. |
| GREEN-ARMYTAGE, Capt. V. B., I.M.S. ... ... | c/o T. Cook & Son, Calcutta. |
| *GREER, W. J. ... ... ... ... ... ... ... ... ... | 19 Gold Tops, Newport, Mon. |
| *GRIFFITHS, CORNELIUS A. ... ... ... ... ... | 35 Newport Road, Cardiff. |
| GRIFFITHS, J. S. ... ... ... ... ... ... ... ... | 20 Redland Park, Bristol. |
| *GROVES, E. W. H., M.D., M.S. Lond. ... ... | 16 Richmond Hill, Clifton. |

### LIST OF SUBSCRIBERS.

| | |
|---|---|
| *Hailes, Clements D. G., M.D., C.M. Ed. ... | 27 Alma Road, Clifton. |
| *Hall, E. G., M.B. Lond. ... ... ... ... ... | 42 Apsley Road, Clifton, Bristol. |
| *Hall, I. Walker, M.D., Ch.B.Vict. ... ... | Linden Gate, Clifton Down Road, Clifton. |
| Handfield-Jones, Montagu, M.D. Lond. ... | 36 Cavendish Square, London, W., 1. |
| *Harris, H. Elwin, B.A., M.B. Cantab. ... ... | 13 Lansdown Place, Clifton. |
| *Harsant, W. H. ... ... ... ... ... ... ... ... | Tower House, Clifton Down Road, Clifton. |
| *Harty, J. P. I., M.B., B.Ch., B.A.O., R.U.I. | 5 Westbourne Place, Clifton. |
| Hawes, C. S. ... ... .. ... ... ... ... ... | St. Andrew's Hospital, Dollis Hill, London, N.W., 2. |
| *Hayman, C. A., M.D. St. And. ... ... ... ... | Kingston Villa, Richmond Hill, Clifton. |
| Hayman, Howard ... ... ... ... .. ... ... | 3 Clifton Park, Clifton. |
| Hele, T. S., M.A., M.B. ... ... ... ... ... | Emmanuel College, Cambridge. |
| *Herapath, C. E. K., M.D. Lond. ... ... ... | 12 Brunswick Square, Bristol. |
| *Herapath, C. K. C. ... ... ... ... ... ... | Stoneleigh, Cotham Park, Bristol. |
| Hill, Hedley, M.D. Durh. ... ... ... ... | 1 Whiteladies Road, Clifton. |
| *Hill, R. A. P., M.D. ... ... ... ... ... ... | St. Keverne, Harrow-on-the-Hill. |
| *Hill, Walter J. ... ... ... ... ... ... ... | The Brow, Clevedon, Som. |
| Hospital, Bristol General (*Secretary*, T. W. Gregg). | |

| | |
|---|---|
| *Iles, A. E., M.B., B.S. Lond. ... ... ... ... | Cotham Lawn, Cotham Park, Bristol. |
| *Imlay, G. A., M.B., C.M. Aberd. ... ... ... | 4 Pembroke Vale, Clifton. |
| Infirmary, Bristol Royal (*Secretary*, W. E. Budgett). | |
| Ingle, C. Durban ... ... ... ... ... ... ... | Somerton, Somerset. |

| | |
|---|---|
| *James, C. W. W. ... ... ... ... ... ... ... ... | 246 Wells Road, Knowle, Bristol. |
| Jamison, A., M.B., B.Ch., M.A. R.U.I. ... ... | Ardenvohu Woodstock Road, Belfast. |
| *Joll, C. A., M.B., M.S. Lond. ... ... ... ... | 21 Wimpole St., London, W.1. |
| *Jones, J. Ellington ... ... ... ... .. ... | 8 Chantry Road Clifton. |
| Joseph, A. Hill, M.D. Lond. ... ... ... ... | Bexhill-on-Sea. |

| | |
|---|---|
| *Kay-Mouat, J. R., M.A. Oxon., M.B., Ch.B. Bristol ... ... ... ... ... ... ... ... ... ... | 5 Gloucester Row, Clifton. |
| *Kerfoot, Stanley J. ... ... ... ... ... ... | 109 East Street, Bedminster, Bristol. |
| *Kerr, R. A., M.B., B.Ch. ... ... ... ... ... | 40 Orients Gardens, Belfast, Ireland. |
| *Kyle, H. G., M.A., M.D., B.Ch. Oxon. ... ... | 31 Westbury Road, Westbury. |

| | |
|---|---|
| *Lace, F. ... ... ... ... ... ... ... ... ... ... | 5 Gay Street, Bath. |
| *Lansdown, R. G. P., M.D., B.S. Durh. ... ... | 39 Oakfield Road, Clifton. |
| *Lavers, Norman, M.D. Brux. ... ... ... ... | Bailbrook House, Bath. |
| *Lavington, C. C., M.B., B.S. Durh. ... ... | 1 Burlington Road, Redland, Bristol. |
| Lendon, A. A., M.D. Lond. ... ... ... ... | 40 Shoot-up-hill, Brondesbury, N.W. |
| *Lees, E. Leonard, M.D., C.M. Ed. ... ... ... | 1 Chesterfield Place, Clifton. |

### LIST OF SUBSCRIBERS.

| | |
|---|---|
| LEIGH, W. W. ... ... ... ... ... ... ... ... | Treharris, Glamorganshire. |
| *LEONARD, R. C., M.D. Durham ... ... ... ... | 4 Royal York Crescent, Clifton. |
| *LEWIS, D. J. ... ... ... ... ... ... ... ... | Glendower, Winscombe, Som. |
| *LINTON, MARION S., M.B. Lond. ... ... ... | 21 Oakfield Road, Clifton. |
| *LLEWELLYN, R. LL. J., M.B. Lond. ... ... ... | 41 Gay Street, Bath. |
| *LONGLEY, J. A. NOEL, M.B., Ch.B. Birm. ... | 124a Redland Road, Bristol. |
| *LUCAS, J. J. S., M.D. Lond. ... ... ... ... ... | 186 Wells Road, Totterdown, Bristol. |

| | |
|---|---|
| MACKINNON, CHARLES, M.B., C.M., M.A. Glasg. | Davaar, Cirencester. |
| MACLEAN, EWEN J., M.D., C.M. Ed.... ... ... | 12 Park Place, Cardiff. |
| MACLEAN, J. CAMPBELL, M.B., C.M. Ed.... ... | Swindon House, Swindon, Wiltshire. |
| *MACLEOD, DONALD, M.B., C.M. Ed. ... ... | 84 Redland Road, Redland, Bristol. |
| *MACWATTERS, J. C. .. ... ... ... ... ... ... | Almondsbury, near Bristol. |
| MARSHALL, ARTHUR L., M.B., B.A. Cantab. ... | Thornleigh, Upper Clapton, London, E.5. |
| *MARTIN, E. F., M.D., C.M. Ed. ... ... ... ... | 7 Royal Terrace, Weston-super-Mare. |
| *MARTYN, G. J. KING, M D., B.C., B.A. Cantab. ... ... ... ... ... ... ... ... ... | 8 Gay Street, Bath. |
| MASON, S. B. ... ... ... ... ... ... ... ... | 12 Park Terrace, Pontypool. |
| MIALL, C. L ... ... ... ... ... ... ... ... ... | Elm Hayes, Paulton, near Bristol. |
| MOLE, HAROLD F. ... ... ... ... ... ... ... ... | 24 College Road, Clifton. |
| *MOORE, C. A., M.B., M.S. Lond. ... ... ... ... | 56 St. Paul's Road, Clifton. |
| MORRIS, L. N., M.B., C.h.B. Bris. ... ... ... | 24 All Hallows Road, Upper Easton, near Bristol. |
| *MORRIS, MARY E. H., M.D. Lond. ... ... ... | 19 Gay Street. Bath. |
| *MORTON, C. A., F.R.C.S. Eng. ... ... ... ... | 14 Vyvyan Terrace, Clifton. |
| MUNDEN, CHARLES ... ... ... ... ... ... ... | Ilminster, Somerset. |
| *MUNRO, J. M. H., D.Sc. Lond. ... ... ... ... | 12 Grosvenor Place, Bath. |
| *MYLES, G T. ... ... ... ... ... ... ... ... | 7 Apsley Road, Clifton. |

| | |
|---|---|
| *NEILD, NEWMAN, M.B., B.Ch. Vict. ... ... ... | 9 Richmond Hill, Clifton. |
| *NEWNHAM, W. H. C., M.B., M.A. Cantab. ... | 3 Lansdown Place, Victoria Square, Clifton. |
| *NICHOLS, F. C., M.B., Ch.B. Bristol ... ... ... | 38 Whiteladies Road, Clifton. |
| *NIXON, J. A., B.A., M.D., B.C. Cantab. ... ... | 7 Lansdown Place, Clifton. |
| *NIXON, J. H., M.D., B.S. Lond. ... ... ... | Morley House, Bath Road, Chippenham. |
| *NOEL-COX, H. L., M.D. Durham ... ... ... | 11 All Saints Road, Clifton. |

| | |
|---|---|
| OPPENHEIMER, SON & CO. LTD. ... ... ... | 179 Queen Victoria Street, London, E.C., 4. |
| *ORMEROD, H. LAWRENCE, M.B., B.Ch. R.U.I. | 2 Henleaze Road, Westbury Park, Bristol. |
| *ORR-EWING, H. J., M.B., B.S. Lond. ... ... | Halmer, South Road, Weston-snper-mare. |

| | |
|---|---|
| PAGE, G. SHEPLEY ... ... ... ... ... ... ... ... | 78 Old Market Street, Bristol. |
| *PARKER, GEORGE, M.D., M.A. Cantab. ... ... | 14 Pembroke Road, Clifton. |
| PARKINSON, Capt. G. S., R.A.M.C. ... ... ... | 25 Cheyne Court, Chelsea, London. |
| *PARSONS, H. F. ... ... ... ... ... ... ... | The Heath, Hazelwood Road, Sneyd Park. |

### LIST OF SUBSCRIBERS.

| | |
|---|---|
| PARSONS, J. H., M.B., B.S., D.Sc. Lond.... ... | 54 Queen Anne Street, London, W. |
| PATERSON, DONALD ROSE, M.D., C.M. Ed. ... | 15 St. Andrew's Crescent, Cardiff. |
| PHILLIPS, C. M., M.D. Brux. ... ... ... ... | 502 Bath Road. |
| *PICKERING, C. F. ... ... ... ... ... ... ... | 13 Berkeley Square, Bristol. |
| *PINEO, E. G. D.... ... ... ... ... ... ... ... | Richmond House, Langford, nr. Bristol. |
| *PORTEOUS, H. B. ... ... ... ... ... ... ... | Newbury House, Weston-super-Mare. |
| POWELL, J. J., M.D. Lond. ... ... ... ... ... | 2 Gloucester Road, Bishopston, Bristol. |
| PRICE, D. T. M.B. Lond. ... ... ... ... ... | Castle Cary, Somerset. |
| *PRICHARD, ARTHUR W. ... ... ... ... ... ... | 6 Rodney Place, Clifton. |
| *PROWSE ARTHUR B., M.D. Lond. ... ... ... | 5 Lansdown Place, Clifton. |

| | |
|---|---|
| RANDOLPH, CHARLES ... ... .. ... .. ... | St. Michael's, Milverton, Somerset. |
| *RATTRAY, J. M., M.D., C.M., M.A. Aberd. ... | Rook Lane House, Frome, Somerset. |
| *RAYNER, D. C., F.R.C.S. Eng. ... ... ... ... | 9 Lansdown Place, Clifton, Bristol. |
| *RICHARDSON, T. A. ... ... ... ... ... ... ... | 10 Clifton Park Road, Clifton. |
| RITCHIE, THOMAS P. ... ... ... ... ... ... | 27 Oakfield Road, Clifton. |
| *ROBERTSON, D., M.B., Ch.B. Edin. ... ... ... | 205 Wells Road, Knowle. |
| *ROGERS, B. M. H., M.D., B.Ch., B.A. Oxon. ... | 14 Mortimer Road, Clifton. |
| *ROLFE, R., B.A., M.B., B.C. Cantab. ... ... | Park House, St. Andrews Road, Avonmouth. |
| *ROSE, F. H. ... ... ... ... ... ... ... ... | Westfield, Portishead, Somerset. |
| *ROSSITER, GEORGE F., M.B. Lond. ... ... ... | Cairo Lodge, Weston-super-Mare. |
| *RUDGE, C. KING ... ... ... ... ... ... ... | 145 Whiteladies Road, Bristol. |
| *RUTHERFORD, J. M., M.B., C.M. Edin., F.R.C.P. | Brislington House, near Bristol. |

| | |
|---|---|
| SCOTT, BATY- ... .. ... ... ... ... ... | 28 Belluton Road, Knowle, Bristol. |
| SEWARD, W. J., M.B. Lond. ... ... ... ... | 15 Chandos Avenue, Oakleigh Park, London, N. |
| *SHORT, A. R., M.D., B.S., B.Sc. Lond. ... ... | Montrose House, Queen's Road, Clifton. |
| *SHORT, L. J., M.D., B.S. Lond., M.D. Bris. ... | Eton Villa, The Shrubbery, Weston super-Mare. |
| *SMITH, G. COCKBURN, M.D. Brux. ... ... ... | 14 South Road, Newton Abbot. |
| SMITH, L. SHINGLETON, B.A., M.B. Cantab.... | Camden Road, Brecon. |
| SMITH, REV. REGINALD, M.A. Cantab. ... ... | The Vicarage, Walpole St. Andrew, Norfolk. |
| SMITH, W. STEELE, M.B. Lond. ... ... ... | 1 Wilbraham Rd., Chorlton-cum-Hardy, Manchester |
| *SMITH, W. ALEXANDER, M.B., M.A. Oxon. ... | 70 Pembroke Road, Clifton. |
| SOCIETY, CARDIFF MEDICAL (*Hon. Lib.*, Queen Street, Cardiff). | |
| SOCIETY, MANCHESTER MEDICAL ... ... ... | Medical Society's Library, The Owens College, Manchester. |
| *STACK E. H. E., B.A., M.B., B.C. Cantab. ... | Arvalee, Clifton Down Road, Clifton. |
| STAPLE, J. D. ... ... ... ... ... ... ... ... | St Martin's, Lyme Regis, Dorset. |
| *STATHAM, R. S. S., M.D. Bristol ... ... ... | 5 Westbourne Place, Clifton. |
| *STOCK, W. S. V., M.B., B.S. Lond. ... ... ... | 1 Mortimer Road, Clifton. |
| *SWAIN, JAMES, M.D., M.S. Lond., C.B. ... ... | 4 Victoria Square, Clifton. |
| *SWAYNE, W. CARLESS, M.D. Lond. ... ... ... | 2 Clifton Park, Clifton. |
| *SYMES, J. O., M.D. Lond. ... ... ... ... ... | 71 Pembroke Road, Clifton. |
| SYMONDS, H. P. ... ... ... ... ... ... ... | 35 Banbury Road, Oxford. |

| | |
|---|---|
| TAYLOR, A. L. | The Royal Infirmary, Bristol. |
| TAYLOR, H. N., M.A. Cantab., M.D. Dublin | Grafton Street, London, W., 1. |
| *TAYLOR, JAMES | 36 Alma Road, Clifton |
| *TAYLOR, J. W., M.D. Bristol | 8 Redcliff Parade West, Bristol. |
| *TAYLOR, S. H. S., B.A. Cantab., M.B., Ch.B.Ed. | Wray Croft, Lacock, Chippenham. |
| *TEMPLE, G. H., M.B., C.M. Ed. | Ailanthus, Bristol Road, Weston-super-Mare. |
| THIN, JAMES | 54 & 55 South Bridge, Edinburgh. |
| *THOMAS, J. D., M.B. Cantab. | Northwoods, Winterbourne. |
| THOMAS, J. LYNN, C.B. | Green Lawn, Pen-y-lan, Cardiff. |
| *THOMAS, J. TUBB, D.P.H. | The Halve House, Trowbridge. |
| *THOMAS, R. E., M.D. B.S. Lond. | c/o E. Thomas, Esq., 14 Melrose Place, Clifton. |
| TOSSWILL, LEONARD R., D.P.H. Oxon. | Hoxton House, Hoxton St., London, N., 1. |
| VACHELL, HERBERT R., M.D., C.M. Aberd. | 18 Newport Road, Cardiff. |
| *VICKERY, W. H. | 1 Trewartha Park, Weston-super-Mare. |
| *WALDO, HENRY, M.D., C.M. Aberd. | 19 Pembroke Road, Clifton. |
| *WALKER, CYRIL H., M.B., B.A. Cantab. | 8 Oakfield Road, Clifton. |
| *WALLACE, JOHN | Lauriston, 56 Knightstone Road, Weston-super-Mare. |
| WALLACE, J. T., M.B., B.Ch., B.A. R.U.I. | 48 Coronation Road, Bedminster, Bristol |
| *WALTERS, C. F. | 5 Mortimer Road, Clifton. |
| *WATERHOUSE, R., M.D.Lond. | 25 Circus, Bath. |
| *WATSON-WILLIAMS, P., M.D. Lond. | 2 Rodney Place, Clifton. |
| *WHICHER, A. H. | 115 Queen's Road, Clifton. |
| WHITE, J. W. | Orchard House, Nailsea. |
| *WHITE, PERCY W. | 37 Whiteladies Road, Clifton. |
| WIGAN, C. L., M.B., B.S. Durham | Clarence House, Portishead. |
| WIGMORE, J., M.D. R. U. I. | 16 Queen Square, Bath. |
| WILLCOX, R. LEWIS | 97 London Road, Salisbury, Wilts. |
| WILLETT, GEORGE G. D. | Keynsham, Somerset. |
| *WILLIAMS, E. C., M.B., B.C., B.A. Cantab. | 159 Whiteladies Road, Clifton. |
| WILLIAMS, H. EGERTON | The Mount, Newport, Mon. |
| WILLIAMS, S. R., M.B. Lond. | Buckfastleigh, Devon. |
| *WILLIAMSON, G. SCOTT | The General Hospital, Bristol. |
| WILLIS, W. M. | 7 Regent Street, Nottingham. |
| *WILLS, W. K., M.B., B.C. Cantab. | 19 Whiteladies Road, Clifton. |
| *WINDSOR-AUBREY, H. W. | 79 Coronation Road, Bristol. |
| *WOOD, DUNCAN | The Porch, Corsham, Wilts. |
| *WOOD, W. V. | Langford, near Bristol. |
| *WRIGHT, A. J., M.B., B.S. Lond. | 14 Victoria Square, Clifton. |
| YOUNG, J., M.D. Glas. | 14 Chantry Road, Clifton. |

Bristol Medico-Chirurgical Journal,
April, 1917.

### EXCHANGE-LIST.

### AFRICA.

### SOUTH AFRICA.

*Twice a month.*

*South African Medical Record.* Cape Town Record Publishing Co.

### AMERICA.

### ARGENTINE REPUBLIC

*Weekly.*

*La Semana Médica.* Buenos Ayres: Junin 845–863.

### CANADA

*Monthly.*

*The Canada Lancet.* Toronto: The Ontario Publishing Co. Ltd.

*The Canadian Medical Association Journal.* Toronto: Morang & Co. Ltd.

### PERU.

*Twice a month.*

*La Crónica Médica.* Lima: Apartado postal, No. 629.

### UNITED STATES.

*Weekly.*

*Boston Medical and Surgical Journal.* Boston, 101 Tremont Street.

*Medical Record.* New York: William Wood & Co.

*Monthly.*

*American Journal of Surgery.* New York: 92 William Street.

*Archives of Pediatrics.* New York. E. B. Treat & Co.

*Buffalo Medical Journal.* Buffalo: 228 Summer Street.

*Bulletin of the Johns Hopkins Hospital.* Baltimore: The Johns Hopkins Press.

*The Bulletin of the Post-Graduate Medical School and Hospital.* New York: 2nd Avenue and 20th Street.

*California State Journal of Medicine.* San Francisco: Butler Building.

*Charlotte Medical Journal.* Charlotte: E. C. Register.

*New York State Journal of Medicine.* New York: 17 West 43rd Street.

*The American Journal of Obstetrics.* New York: William Wood & Co.

*The American Journal of Ophthalmology.* St. Louis: 316 Metropolitan Building.

*The Therapeutic Gazette.* Detroit: E. G. Swift.

*Quarterly.*

*The Alienist and Neurologist.* St. Louis: 3858 West Pine Boulevard.

*Irregularly.*

*The Journal of Experimental Medicine.* New York The Rockefeller Institute for Medical Research.

*The Journal of Medical Research.* Boston: 240 Longwood Avenue.

*Yearly.*

*Smithsonian Report.* Washington: Government Printing Office.

*Studies from the Department of Pathology of the College of Physicians and Surgeons—Columbia University.* New York: 437 West 59th Street.

*Transactions of the American Association of Obstetricians and Gynecologists.* Philadelphia: William J. Dornan.

*Transactions of the American Dermatological Association.* New York: The Grafton Press.

*Transactions of the American Laryngological Association.* New York: The McConnell Printing Co.

*Transactions of the American Ophthalmological Society.* Hartford: The Case, Lockwood & Brainard Co.

*Transactions of the American Pediatric Society,* New York: E. B. Treat & Co.

*Transactions of the American Surgical Association* Philadelphia: William J. Dornan.

*Transactions of the Association of American Physicians.* Philadelphia: William J. Dornan.

*Transactions of the College of Physicians of Philadelphia.* Philadelphia: William J. Dornan.

### ASIA

### INDIA.

*Monthly.*

*The Antiseptic.* Madras: U. Rama Rau.

*The Indian Medical Gazette.* Calcutta: Thacker Spink & Co.

*Practical Medicine* Delhi: Egerton Street.

### JAPAN.

*Monthly.*

*The Sei-I-Kwai Medical Journal.* Tokyo: Charity Hospital Medical College.

### PHILIPPINE ISLANDS.

*Bi-monthly.*

*The Philippine Journal of Science* Manila: Bureau of Printing.

### EXCHANGE-LIST (*continued*).

### AUSTRALASIA.

### NEW ZEALAND.

*Quarterly.*

*New Zealand Medical Journal.* Wellington: "The Dominion" Office.

### EUROPE.

### AUSTRIA.

*Weekly.*

**Wiener klinische Wochenschrift.* Vienna: Wilhelm Braumüller.

### BELGIUM.

*Monthly.*

**Bulletin de l'Académie royale de Médecine de Belgique.* Brussels: J. Goemaere.

### BRITISH ISLES.

*Weekly.*

*British Medical Journal.* London: 429 Strand.

*Medical Press and Circular.* London: A. A. Tindall.

†*The Clinical Journal.* London: The Medical Publishing Company, Limited.

*The Hospital.* London: The Scientific Press (Limited).

*The Lancet.* London: 423 Strand.

*The Sanitary Record.* London: The Sanitary Publishing Co., Ltd.

*Fortnightly.*

*The Journal of Tropical Medicine.* London John Bale, Sons & Danielsson, Ltd.

*Monthly.*

*Edinburgh Medical Journal.* Edinburgh: Wm. Green & Sons.

*Public Health.* London: The Society of Medical Officers of Health.

*The Birmingham Medical Review.* Birmingham: Percival Jones, Ltd.

‡*The British Journal of Dermatology.* London: H. K. Lewis.

*The Glasgow Medical Journal.* Glasgow: Alex. Macdougall.

*The Journal of Laryngology, Rhinology, and Otology.* London: Adlard & Son.

*The Medical Review.* London: 66 Finsbury Pavement.

*The Polyclinic.* London: John Bale, Sons & Danielsson, Ltd

*The Practitioner.* London: Howard Street, Strand.

*The Prescriber.* Edinburgh: 137 George Street.

*The Proceedings of the Royal Society of Medicine.* London: Longmans, Green & Co

*Quarterly.*

*Journal of Mental Science.* London: J. & A. Churchill

*The Caledonian Medical Journal.* Glasgow: Alex Macdougall.

*The West London Medical Journal.* London: Adlard & Son.

*Yearly during War.*

*The Liverpool Medico-Chirurgical Journal.* Liverpool: Medical Institution.

*Yearly.*

*Guy's Hospital Reports.* London: J. & A. Churchill.

*King's College Hospital Reports.* London: Adlard & Son.

*Ophthalmological Transactions.* London: J. & A Churchill.

*St. Bartholomew's Hospital Reports.* London Smith, Elder & Co.

*St. Thomas's Hospital Reports.* London: J. & A. Churchill.

*The Medical Annual.* Bristol: John Wright & Sons Ltd.

*The Medical Society's Transactions.* London: Harrison & Sons.

*The Transactions of the Edinburgh Obstetrical Society.* Edinburgh: Oliver and Boyd.

*The Westminster Hospital Reports.* London: Henry J. Glaisher.

*Transactions of the Edinburgh Medico-Chirurgical Society.* Edinburgh: James Thin.

*Transactions of the Royal Academy of Medicine in Ireland.* Dublin: John Falconer.

### DENMARK.

*Weekly.*

*Hospitalstidende.* Copenhagen: Jacob Lund.

### FRANCE.

*Three times a week.*

**Gazette des Hôpitaux.* Paris: 49 Rue St.-André des-Arts.

*Weekly.*

*Bulletin de l'Académie de Médecine.* Paris: Masson et Cie

**Gazette des Hôpitaux de Toulouse.* Toulouse: 13 Rue Ste-Ursule.

*Gazette hebdomadaire des Sciences médicales de Bordeaux.* Bordeaux: 8 Rue Paul-Bert.

**L'Écho médical du Nord.* Lille: 128 Boulevard de la Liberté.

*Le Progrès médical.* Paris: 41 Rue des Ecoles.

*Revue hebdomadaire de Laryngologie, d'Otologie et de Rhinologie.* Bordeaux: G. Gounouilhou.

* Discontinued owing to the war. † Published monthly during war.
‡ Published quarterly during war.

### EXCHANGE-LIST (*continued*).

FRANCE—(continued).

*Fortnightly.*

**Gazette des Praticiens.* Lille: Rue des Guinguettes, 18.

*Twice a month.*

**Gazette de Gynécologie.* Paris: 69 Rue d'Amsterdam.

**Revue de Thérapeutique médico-chirurgicale.* Paris: Félix Alcan.

*Monthly.*

**Journal d'Hygiène.* Paris: 79 Avenue de Wagram.

**Revue générale d'Ophtalmologie.* Paris: Masson et Cie.

*Journal de Médecine de Bordeaux.* Bordeaux: 11 rue Guirande.

GERMANY.

*Weekly*

**Zentralblatt für Chirurgie.* Leipzig: J. A. Barth.

**Zentralblatt für Gynäkologie.* Leipzig: J. A. Barth.

**Zentralblatt für innere Medicin.* Leipzig: J. A. Barth.

**Münchener medicinische Wochenschrift.* Munich: J. F. Lehmann.

GREECE.

*Monthly.*

*Ιατρική πρόοδος.* Syra: Renieri Brindesi.

*La Grèce médicale.* Syra: Renieri Brindesi.

HOLLAND.

*Weekly.*

**Nederlandsch Tijdschrift voor Geneeskunde.* Amsterdam: F. van Rossen.

ITALY.

*Monthly.*

*La Medicina Practica.* Naples.

*Irregular.*

*Archivio di Ortopedia.* Milan: Via San Calimero, 31.

NORWAY.

*Monthly.*

*Norsk Magazin for Lægevidenskaben.* Christiania: Steen'ske Bogtrykkeri.

RUSSIA.

*Weekly.*

**Medycyna.* Warsaw: Niecala, 7.

*Twice a month.*

**St. Petersburger medicinische Zeitschrift.* St. Petersburg: K. L. Ricker.

*Monthly.*

*Finska Läkaresällskapets Handlingar.* Helsingfors: Mercators Tryckeri.

SPAIN.

*Weekly*

*El Siglo Médico.* Madrid: Magdalena 36.

SWEDEN.

*Bi-monthly.*

*Nordiskt Medicinskt Arkiv.* Stockholm: P. A. Norstedt & Söner.

TURKEY.

*Monthly.*

**Gazette médicale d'Orient.* Constantinople: "Levant Herald."

* Discontinued owing to the war.

---

Books for Review and Exchange Journals should be addressed: The Assistant-Editor, *Bristol Medico-Chirurgical Journal*, Medical Library, The University, Bristol

### Bristol Medico-Chirurgical Journal

*Established in 1883 as a Journal of the Medical Sciences for the West of England and South Wales.*

Published Quarterly (Demy 8vo, price 1/6), under the auspices of the Bristol Medico-Chirurgical Society.

### Terms for Advertisements.

| | £ | s. | d. |
|---|---|---|---|
| Whole Page ... ... ... ... | 1 | 1 | 0 |
| Half Page ... ... ... ... ... | 0 | 12 | 6 |
| Quarter Page ... ... ... ... | 0 | 7 | 6 |

Insets (2 or 4 pages), 20/-

Discount of 5 per cent. will be allowed if payment is made at the time of ordering.

Terms for special positions upon application.

*Orders to be sent to the Publishers—*

J. W. ARROWSMITH LTD., Quay Street, Bristol.

